# Detection of HER2 expression using ^99m^Tc-NM-02 nanobody in patients with breast cancer: a non-randomized, non-blinded clinical trial

**DOI:** 10.1186/s13058-024-01803-y

**Published:** 2024-03-08

**Authors:** Lingzhou Zhao, Yan Xing, Changcun Liu, Shaofei Ma, Wenhua Huang, Zhen Cheng, Jinhua Zhao

**Affiliations:** 1 Department of Nuclear Medicine, Shanghai General Hospital, Shanghai Jiao Tong University School of Medicine, No. 100, Haining Road, Hongkou District, Shanghai, 200080 China; 2 Department of Pathology, Shanghai General Hospital, Shanghai Jiao Tong University School of Medicine, No. 100, Haining Road, Hongkou District, Shanghai, 200080 China; 3 Nanomab Technology Limited, No. 333, North Chengdu Road, Jingan District, Shanghai, 200041 China; 4 State Key Laboratory of Drug Research, Molecular Imaging Center, Shanghai Institute of Materia Medica, Chinese Academy of Sciences, No. 555, Zuchongzhi Road, Pudong New District, Shanghai, 201203 China; 5 Shandong Laboratory of Yantai Drug Discovery, Bohai Rim Advanced Research Institute for Drug Discovery, No. 198, Binhai East Road, High-Tech District, Yantai, 264000 Shandong China

**Keywords:** HER2, Breast cancer, Nanobody, SPECT/CT imaging, ^99m^Tc

## Abstract

**Background:**

^99m^Tc radiolabeled nanobody NM-02 (^99m^Tc-NM-02) is a novel single photon emission computed tomography (SPECT) probe with a high affinity and specificity for human epidermal growth factor receptor 2 (HER2). In this study, a clinical imaging trial was conducted to investigate the relationship between ^99m^Tc-NM-02 uptake and HER2 expression in patients with breast cancer.

**Methods:**

Thirty patients with pathologically confirmed breast cancer were recruited and imaged with both ^99m^Tc-NM-02 SPECT/computed tomography (CT) and ^18^F-fluorodeoxyglucose (^18^F-FDG) positron emission tomography (PET)/CT. According to the treatment conditions before recruitment, patients were divided into two groups, the newly diagnosed group (*n* = 24) and the treated group (*n* = 6). The maximal standard uptake value (SUV_max_) of ^18^F-FDG and SUV_max_ and mean SUV (SUV_mean_) of ^99m^Tc-NM-02 in the lesions were determined to analyze the relationship with HER2 expression.

**Results:**

No meaningful relationship was observed between ^18^F-FDG uptake and HER2 expression in 30 patients with breast cancer. ^99m^Tc-NM-02 uptake was positively correlated with HER2 expression in the newly diagnosed group, but no correlation was observed in the treated group. ^99m^Tc-NM-02 uptake in HER2-positive lesions was lower in those with effective HER2-targeted therapy compared with the newly diagnosed group. ^99m^Tc-NM-02 SPECT/CT detected brain and bone metastases of breast cancer with a different imaging pattern from ^18^F-FDG PET/CT. ^99m^Tc-NM-02 showed no non-specific uptake in inflamed tissues and revealed intra- and intertumoral HER2 heterogeneity by SPECT/CT imaging in 9 of the 30 patients with breast cancer.

**Conclusions:**

^99m^Tc-NM-02 SPECT/CT has the potential for visualizing whole-body HER2 overexpression in untreated patients, making it a promising method for HER2 assessment in patients with breast cancer.

**Trial registration:**

NCT04674722, Date of registration: December 19, 2020.

**Supplementary Information:**

The online version contains supplementary material available at 10.1186/s13058-024-01803-y.

## Background

Human epidermal growth factor receptor 2 (HER2) is overexpressed in approximately 15–20% of breast cancers [1]. HER2-positive breast cancer is associated with more tumor aggressiveness, a higher recurrence rate, and shorter overall survival than HER2-negative breast cancer [2]. Over the past 20 years, the clinical application of HER2-targeted therapy has changed breast cancer diagnosis and treatment patterns and greatly improved the prognosis of patients with HER2-positive breast cancer [3, 4]. The response to such therapy depends on the patient’s HER2 status; thus, accurate detection of HER2 expression level in patients with breast cancer is important.

Current clinical approaches to determine HER2 status include immunohistochemistry (IHC) and fluorescence in situ hybridization (FISH) of tumor tissues, usually obtained from a biopsy or operation [5]. However, because of the spatial and temporal heterogeneity of HER2 expression in breast cancer, this invasive method only detects HER2 expression in a small portion of tumor tissues, resulting in false-negative results and failure to systemically reflect and dynamically monitor HER2 status during the disease course [6, 7]. This clinical problem has prompted the development of novel imaging strategies to evaluate the expression and distribution of HER2 comprehensively and accurately [8, 9]. 

Compared to biopsy-based methodologies, radionuclide molecular imaging has unique advantages for providing a non-invasive and comprehensive evaluation of HER2 expression through HER2-targeted molecular probes, including visualization of whole-body HER2 expression, detection of intra- and intertumoral HER2 heterogeneity, and determination of HER2 expression changes over time via repetitive evaluation [10, 11]. Multiple probes have been developed for HER2-targeted molecular imaging, including antibody-, affibody-, nanobody-, and peptide-based probes [12–14]. Among them, nanobodies (i.e., single-domain antibodies), naturally derived from heavy-chain-only antibodies, have been proven to be superior candidates for developing HER2-targeted molecular probes. These probes are characterized by high specificity, deep tumor penetration capability, and rapid blood clearance [15–17]. Based on their distinctive properties, several radionuclide-labeled HER2-targeted nanobodies have been reported, indicating a high potential for clinical application [18–23].

In our previous study, an HER2-targeted nanobody, NM-02, was screened and selected from an established phage display library [23]. NM-02 had a high binding affinity and specificity for HER2 proteins. In the first-in-human study, ^99m^Tc radiolabeled nanobody NM-02 (^99m^Tc-NM-02) showed excellent safety, favorable biodistribution, and distinct imaging characteristics in 10 patients with breast cancer. Based on the results of that study, we recruited 30 patients with breast cancer for a clinical trial to further evaluate the potential application of ^99m^Tc-NM-02 as a single photon emission computed tomography (SPECT) probe for HER2 expression assessment. The primary objective of this study was to assess the relationship between patient tumor ^99m^Tc-NM-02 uptake and HER2 results obtained by IHC and FISH. The secondary objective was to visualize the heterogeneity of HER2 expression using ^99m^Tc-NM-02 in patients with breast cancer.

## Methods

This study was approved by the Ethics Committee of Shanghai General Hospital (2020[84]), and all participants signed an informed consent form. The clinical trial is registered at ClinicalTrials.gov (NCT04674722).

In this non-randomized and non-blinded clinical trial, the preparation and quality control of ^99m^Tc-NM-02, patient selection, and safety assessment were performed according to the previously described protocols [23]. Thirty patients with pathologically confirmed breast cancer were recruited and underwent both ^99m^Tc-NM-02 SPECT/computed tomography (CT) and ^18^F-fluorodeoxyglucose (^18^F-FDG) positron emission tomography (PET)/CT imaging and a follow-up after one week (Supplementary Fig. S1). IHC and FISH were performed on preoperative samples by a pathologist bind to the clinical data. The HER2 expression level was determined by IHC using DAKO HercepTest and scored as 0, 1+, 2+, or 3+. HER2 gene amplification was confirmed by FISH. The samples were defined as HER2-positive (IHC 3 + or IHC 2 + and FISH-positive) and HER2-negative (IHC 0, or IHC 1+, or IHC 2 + but FISH-negative). If subsequent surgery was performed, postoperative pathology was also evaluated and compared with the preoperative result. During the follow-up, effective therapy was assessed by the physicians from the department of breast-thyroid surgery according to the imaging changes in lesions before and after treatment, such as the reduction or disappearance of lesions.

^99m^Tc-NM-02 SPECT/CT and ^18^F-FDG PET/CT were performed according to previously reported protocols [23, 24]. Routine whole-body SPECT and local SPECT/CT images were obtained at 1 and 2 h post-injection using the GE Discovery NM670 SPECT/CT system (GE Healthcare, USA). ^18^F-FDG PET/CT images were obtained 1 h post-injection from the skull base to the mid-thigh using a Philips Vereos PET/CT system (Philips, Netherlands). The maximal and mean standard uptake values (SUV_max_ and SUV_mean_) of ^99m^Tc-NM-02 in tumor lesions were calculated using the Q.Metrix software (GE Healthcare, USA). The region of interest was drawn on the attenuation-corrected PET/CT images around the suspected lesion sites to determine the SUV_max_ of ^18^F-FDG. After ^18^F-FDG PET/CT and ^99m^Tc-NM-02 SPECT/CT, two experienced nuclear medicine physicians independently performed visual interpretation and quantitative analysis of all lesions in 30 patients (^18^F-FDG for SUV_max_ and ^99m^Tc-NM-02 for SUV_mean_ and SUV_max_). Visual interpretation was performed through a visual comparison of ^99m^Tc-NM-02 accumulation between the lesion and surrounding normal tissue. Imaging was considered positive by visual interpretation when ^99m^Tc-NM-02 uptake in any lesion was greater than that in the background, corresponding to the lesion identified on CT imaging. Otherwise, it was considered negative.

Data are presented as the mean ± standard deviation and were analyzed using IBM SPSS software (version 24.0). Mean values were compared using Student’s *t* test. Correlations were tested using Spearman’s rank. Statistical significance was set at *P* < 0.05 (∗ < 0.05, ∗∗ < 0.01, and ∗∗∗ < 0.001).

## Results

### Patients

Between January and August 2021, 30 patients with pathologically confirmed breast cancer completed the study protocol. On-going treatment was not an exclusion criterion. Twenty-four patients who had not received any antitumor treatment prior to ^99m^Tc-NM-02 imaging were newly diagnosed; the remaining six had received multiple cycles of HER2-targeted therapy or chemotherapy before recruitment. According to their treatment status before enrollment, the patients were divided into newly diagnosed (*n* = 24) or treated (*n* = 6) groups. Owing to the double primary lesions with different HER2 expression levels found in two patients (patient BR014 and patient BR018), 32 primary tumors were identified in 30 patients. Preoperative pathological results of the primary lesions showed the following scores for HER2 IHC: 0 in 6 patients, 1 + in 10 patients, 2 + in 6 patients, and 3 + in 10 patients. Four patients were unsuitable for surgery due to multiorgan metastases. Postoperative pathological results were obtained in 26 patients, and half were confirmed to have metastatic lesions. Lymph node (17 cases), bone (3 cases), cerebral (1 case), and liver (2 cases) metastasis were found. The characteristics of the 30 patients are detailed in Supplementary Table S1.

Although a preliminary clinical study has proven the safety of ^99m^Tc-NM-02 for SPECT/CT imaging, monitoring adverse drug reactions remains an essential objective of this study [23]. No drug-related adverse reactions or symptoms were observed during the whole study, further supporting the safety of ^99m^Tc-NM-02 in vivo.

### ^18^F-FDG PET/CT and ^99m^Tc-NM-02 SPECT/CT imaging

^18^F-FDG PET/CT and ^99m^Tc-NM-02 SPECT/CT images were carefully interpreted and analyzed (Supplementary Table S2). ^18^F-FDG PET/CT showed a good diagnostic performance for both primary and metastatic lesions in all patients, with SUV_max_ ranging from 2.3 to 45.7 and no relationship with HER2 IHC scores or status (*P* > 0.05). ^18^F-FDG PET/CT identified 32 primary lesions in 30 patients, bone metastasis in 3 patients, liver metastasis in 2 patients, cerebral metastasis in 1 patient, and lymph node metastasis in 25 patients, of whom 17 were confirmed by the postoperative pathological results. ^99m^Tc-NM-02 SPECT/CT showed positive imaging results in the primary lesions of 22 patients and metastases in 12 patients. Except for one patient with a multifocal primary lesion (patient BR030), no significant uptake of ^99m^Tc-NM-02 was found in the primary lesions of the newly diagnosed patients with HER2 IHC 0, whereas SPECT/CT showed different levels of ^99m^Tc-NM-02 uptake in the primary and metastatic lesions of the other patients in the newly diagnosed group. Notably, the primary, and particularly, metastatic lesions in the treated group had low ^99m^Tc-NM-02 uptake. Whole-body SPECT images of ^99m^Tc-NM-02 primarily showed accumulation in the liver and kidneys with mild uptake in the spleen, intestines, and glandular tissues, but only background levels in other organs where primary tumors and metastases typically occurred (Supplementary Fig. S2). This biodistribution pattern is consistent with the preliminary clinical study [23]. 

## Relationship between ^99m^Tc-NM-02 uptake and HER2 expression

**Fig. 1:**
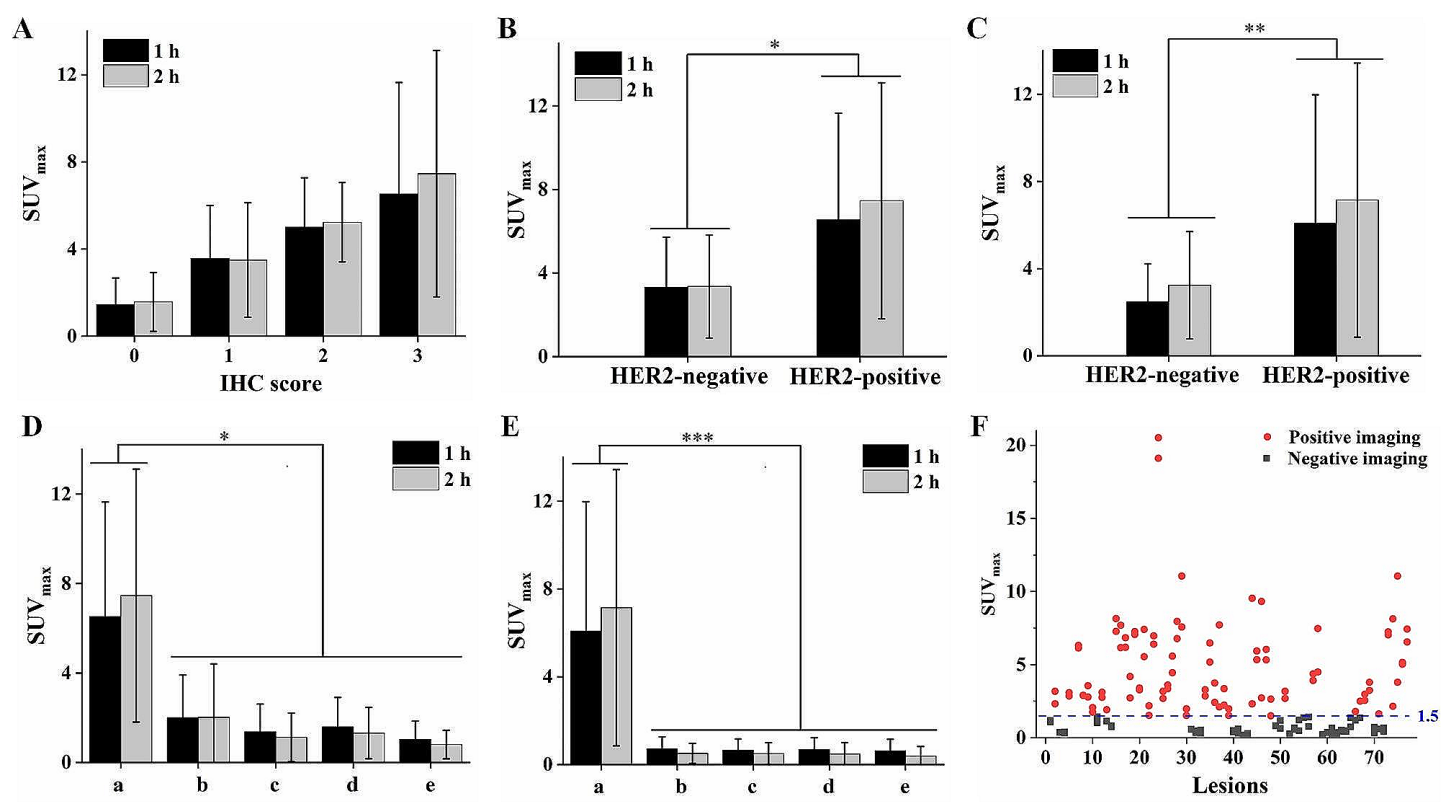
Relationship between ^99m^Tc-NM-02 SUV_max_ and HER2 IHC scores (**A**) and HER2 status (**B**) in the primary lesions of the newly diagnosed group. Relationship between ^99m^Tc-NM-02 SUV_max_ and HER2 status in the metastases of the newly diagnosed group (**C**). Relationship between ^99m^Tc-NM-02 SUV_max_ and HER2 status in the primary lesions (**D**) and metastases (**E**) of the treated group; a: seven HER2-positive patients in the newly diagnosed group, b: six patients in the treated group, c: five patients with high HER2 expression (HER2 2 + and 3+) in the treated group, d: four HER2-positive patients in the treated group, e: three HER2-positive patients with effective HER2-targeted therapy in the treated group. The ^99m^Tc-NM-02 SUV_max_ and visual interpretation in all lesions at 1 and 2 h post-injection (**F**). The red line indicates a proposed threshold at SUV_max_ = 1.5 for discriminating HER2-positive and HER2-negative imaging

In the newly diagnosed group, the SUV_max_ of the primary lesions had a positive correlation with the HER2 IHC scores at 1 h (r^2^ = 0.992, *P* = 0.004) and 2 h post-injection (r^2^ = 0.996, *P* < 0.001), indicating that ^99m^Tc-NM-02 uptake increased as the HER2 IHC scores increased (from 0 to 3+, Fig. 1A). Moreover, similar relationships were found between the SUV_max_ of primary lesions (Fig. 1B) and metastases (Fig. 1C) and HER2 status in the newly diagnosed group. ^99m^Tc-NM-02 uptake in the HER2-positive lesions was significantly higher than that in the HER2-negative lesions (*P* < 0.05). Conversely, the SUV_max_ of the primary lesions (Fig. 1D) and metastases (Fig. 1E) in the treated group was low and had no relationship with HER2 IHC scores or status. The pathological results of the six patients in the treated group showed HER2 IHC 0 in one patient, 2 + in two patients, and 3 + in three patients (Supplementary Table S1). For a comprehensive comparison with the seven HER2-positive patients in the newly diagnosed group (subgroup a), multiple subgroups (b–e) were set in the treated group, which contained all the patients in the treated group, five patients with high HER2 expression (HER2 2 + and 3+), four HER2-positive patients, and three HER2-positive patients with effective HER2-targeted therapy, respectively. Unsurprisingly, subgroup a had significantly higher ^99m^Tc-NM-02 uptake than the other subgroups, and subgroup e had the lowest uptake, regardless if the lesions were primary or metastatic. The lower ^99m^Tc-NM-02 uptake in the lesions was probably related to the influence of treatment. However, more data from a larger sample size are needed to confirm this hypothesis. Moreover, the relationship between SUV_mean_ of lesions and HER2 expression was analyzed, and similar results were acquired (Supplementary Fig. S3). In addition, a comparative analysis between the visual interpretation results of the lesions and their SUV_max_ showed good consistency when the SUV_max_ was 1.5 as the positive imaging threshold (Fig. 1F). It should be noted that delayed imaging is necessary for lesions with suspected ^99m^Tc-NM-02 uptake. For example, the visual interpretation of patient BR017 (HER2 3+) seemed to be negative at 1 h post-injection but was positive at 2 h, which was consistent with the corresponding SUV_max_ (1.49 vs. 2.19, Supplementary Fig. S4).

## Brain and bone metastases imaging

**Fig. 2:**
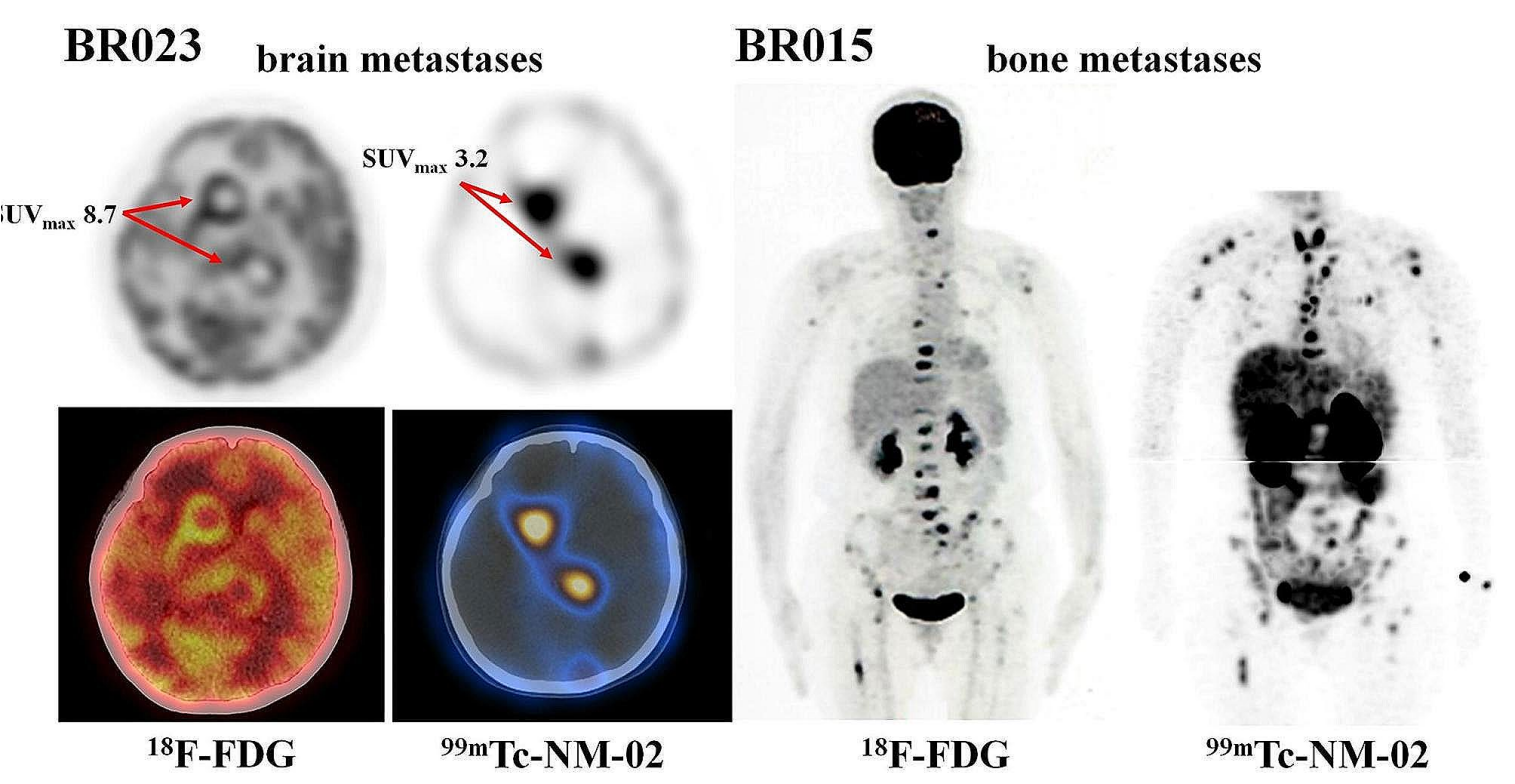
^18^F-FDG PET/CT and ^99m^Tc-NM-02 SPECT/CT of brain (patient BR023) and bone (patient BR015) metastases at 1 h post-injection. Patient BR023 was initially diagnosed with HER2-positive breast cancer on the right side (HER2 IHC 3+) with a clinical stage of T3N3M1. Metastases were found in the lymph node, bone, liver, and brain. This patient had undergone treatment with four cycles of trastuzumab–pertuzumab–docetaxel before imaging. ^18^F-FDG and ^99m^Tc-NM-02 showed low-level uptake in all lesions except brain metastases. Patient BR015 was initially diagnosed with HER2-negative breast cancer on the left side (HER2 IHC 0) with a clinical stage of T2N3M1. Metastases were found in the lymph node and bone. Compared to ^18^F-FDG PET/CT, ^99m^Tc-NM-02 SPECT/CT had a high uptake in more lesions, suggesting potential HER2 positivity

^99m^Tc-NM-02 SPECT/CT detected brain and bone metastases of breast cancer with a different uptake pattern from ^18^F-FDG PET/CT imaging (Fig. 2). In patient BR023, ^18^F-FDG PET/CT indicated low uptake in the central region of the brain metastasis and accumulation in the lesion’s periphery “ring” state. At the same time, ^99m^Tc-NM-02 SPECT/CT displayed uniform uptake in the brain lesion. This finding suggested the inconsistency between HER2 expression and glycolysis in brain metastases, and HER2 expression may be high in lesions with low glycolysis. In patient BR015, ^18^F-FDG PET/CT and ^99m^Tc-NM-02 SPECT/CT in patients with bone metastasis matched well in lesion detection, but the degree of uptake was inconsistent. Compared with ^18^F-FDG PET/CT, ^99m^Tc-NM-02 SPECT/CT showed high uptake in more lesions, suggesting potential HER2 positivity. Notably, this patient’s continued progression of bone metastases during follow-up after nine months of endocrine therapy was consistent with the highly invasive and metastatic nature of breast cancer cells with high HER2 expression.

## No non-specific uptake of ^99m^Tc-NM-02 in inflammation tissues

**Fig. 3:**
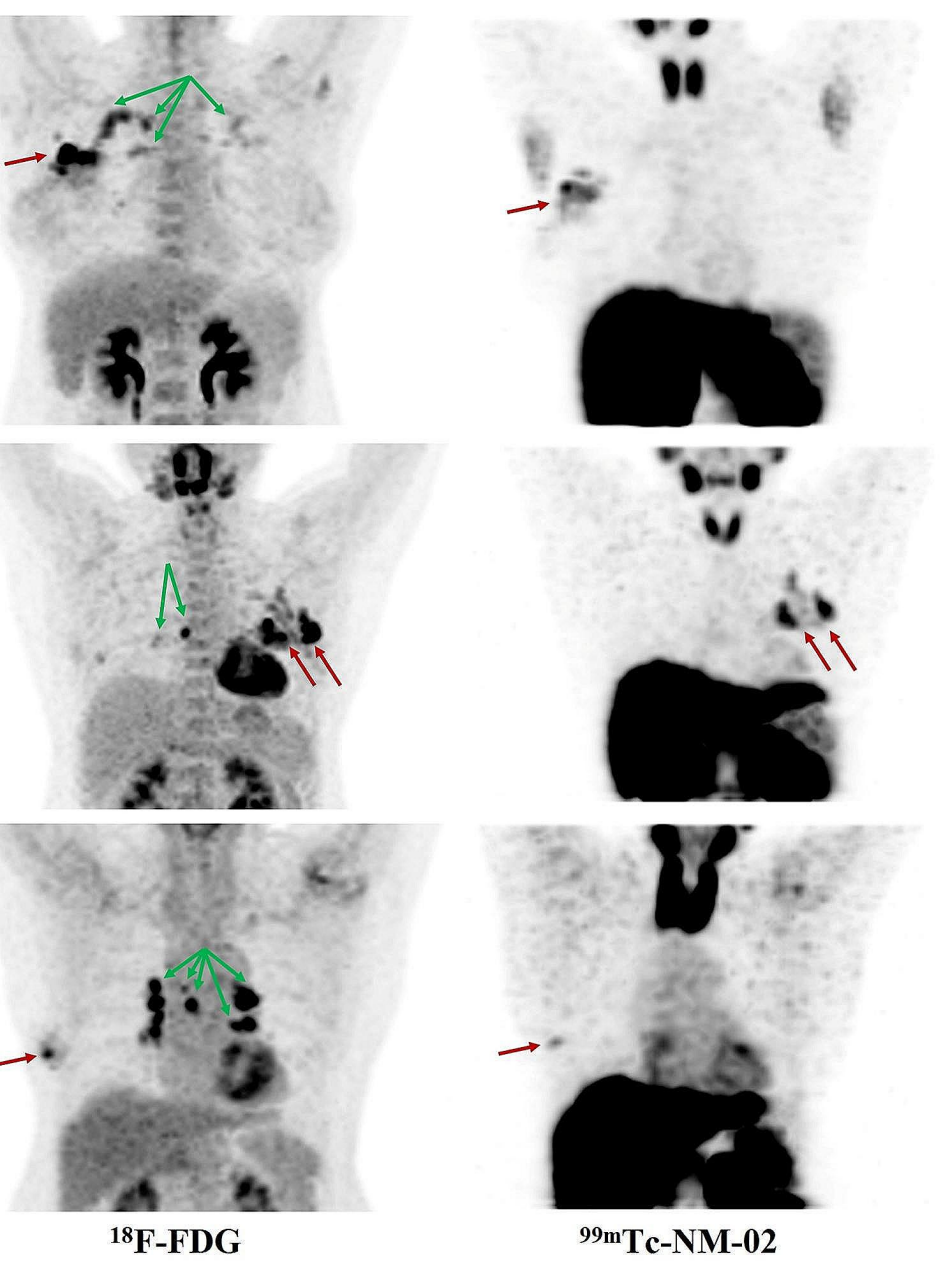
^18^F-FDG PET/CT and ^99m^Tc-NM-02 SPECT/CT imaging of three patients with breast cancer (red arrows) and chest inflammation (green arrows) at 1 h post-injection

On CT before enrollment, three patients with breast cancer (BR011, BR018, and BR027) were diagnosed with chest inflammation, including multiple inflammations in the whole lung and multiple lymphadenites in the mediastinum. ^18^F-FDG PET/CT showed high uptake in these lesions and chest inflammation sites, while a noticeable ^99m^Tc-NM-02 uptake in SPECT/CT was only found in the tumor, not in inflammation tissues (Fig. 3). In addition, subsequent anti-inflammatory therapy was effective for these patients.

## Heterogeneity of HER2 expression by ^99m^Tc-NM-02 SPECT/CT imaging

**Fig. 4:**
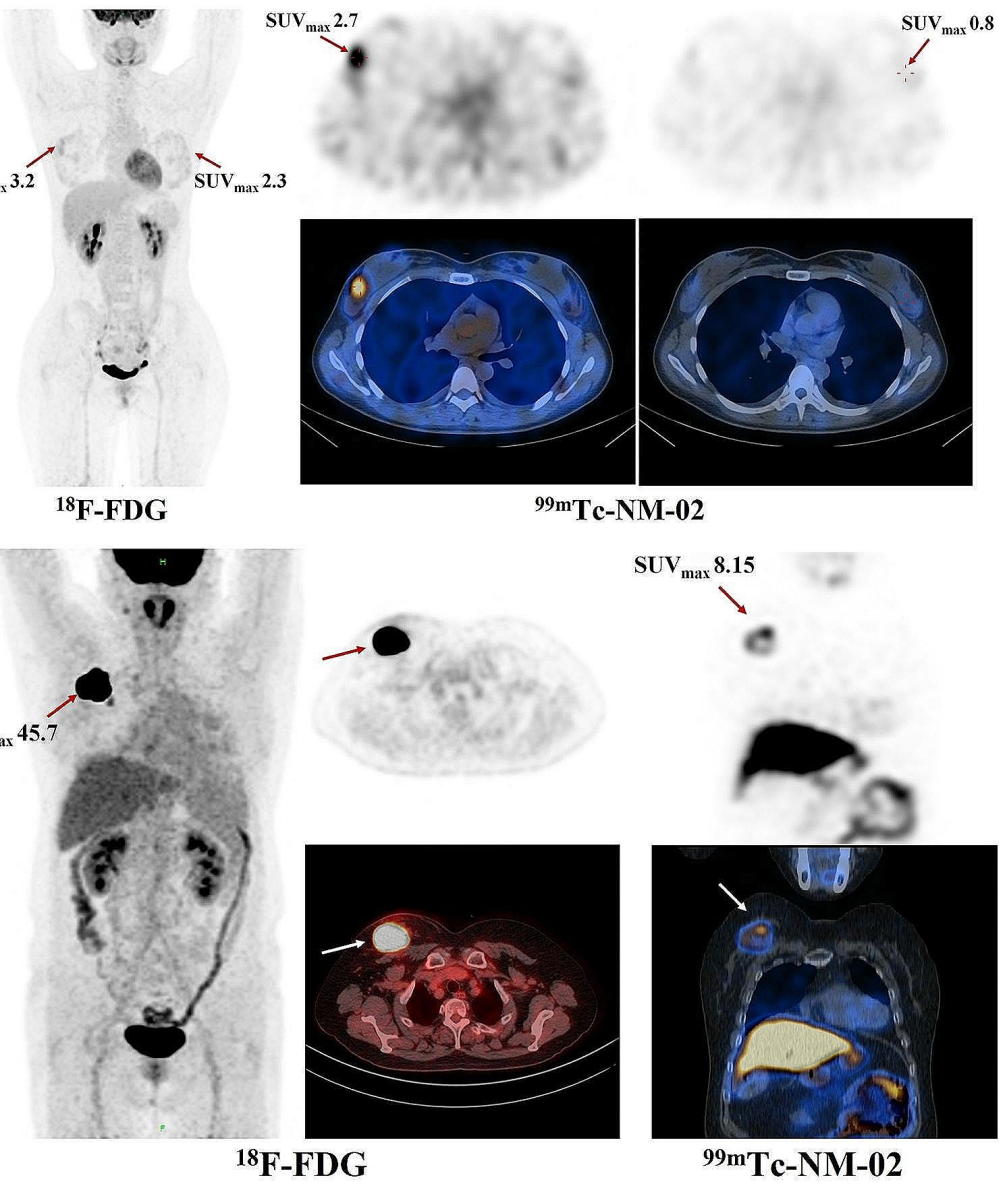
^18^F-FDG PET/CT and ^99m^Tc-NM-02 SPECT/CT of Patient BR014 and Patient BR037 at 1 h post-injection

Heterogeneity of HER2 expression by ^99m^Tc-NM-02 SPECT/CT imaging was defined as heterogeneous tracer uptake in the lesions, which could be summarized as follows: uneven uptake in a single lesion, inconsistent uptake among multiple lesions, and heterogeneous uptake between primary and metastatic lesions. ^99m^Tc-NM-02 SPECT/CT revealed distinct HER2 heterogeneity in 9 of the 30 patients. Taking patient BR014 as a typical example, this woman was diagnosed with bilateral primary breast lesions with different HER2 expression levels. As shown in Fig. 4, bilateral primary breast lesions were observed. The HER2 IHC results for the right and left breast lesions were 2 + and 0, respectively, corresponding to the SUV_max_ (2.7 vs. 0.8). Intertumoral heterogeneity of HER2 expression was not only confirmed by pathology but also visualized by ^99m^Tc-NM-02 SPECT/CT imaging.

Similarly, intratumoral heterogeneity was observed in patient BR037. This patient had homogeneous ^18^F-FDG uptake with a high SUV_max_ of 45.7, whereas heterogeneous ^99m^Tc-NM-02 accumulation was observed in the primary lesion. The low uptake of ^99m^Tc-NM-02 in the central region of the lesion implied low HER2 expression, which was consistent with the result of HER2 IHC 1 + from the needle biopsy. In addition, heterogeneity in the remaining seven patients is shown in Supplementary Fig. S5, including uneven uptake of ^99m^Tc-NM-02 within the primary lesions, inconsistent uptake of ^99m^Tc-NM-02 in multiple unilateral lesions, or heterogeneous ^99m^Tc-NM-02 uptake between primary and metastatic lesions. Moreover, the pathological results of HER2 were inconsistent in six patients before and after treatment, indicating that antitumor treatment might change the expression of HER2 in patients with breast cancer (Supplementary Table S3).

## Potential value of ^99m^Tc-NM-02 SPECT/CT in therapeutic evaluation of HER2-targeted therapy

After completing the clinical trial, the follow-up treatment data of all 30 patients with breast cancer were carefully collected (Supplementary Fig. S6). The potential role of ^99m^Tc-NM-02 in evaluating the therapeutic effect of HER2-targeted therapy was investigated. After treatment, 18 (18/20) HER2-negative patients had a complete response. Two patients, BR015 and BR030, had progressive disease. BR015 had multiple bone metastases. ^99m^Tc-NM-02 SPECT/CT showed obvious uptake in these locations, suggesting the presence of potential HER2-positive lesions (Fig. 2). After several cycles of endocrine and anti-bone metastasis therapy, CT results suggested disease progression. Patient BR030 had multiple lesions in the left breast, and only the larger lesion with high ^18^F-FDG but low ^99m^Tc-NM-02 uptake was biopsied to determine the HER2 status (HER2 IHC 0). In contrast, the ^99m^Tc-NM-02 uptake in a smaller lesion was high, suggesting potential HER2 positivity (Supplementary Fig. S7). After several cycles of postoperative adjuvant chemotherapy, ^18^F-FDG PET/CT results suggested disease progression with lymph node and bone metastasis.

Except for two patients who received surgical treatment, 7 of the 10 HER2-positive patients who received HER2-targeted therapy benefited from the treatment, and one experienced a treatment failure (patient BR023, Supplementary Fig. S8). Before enrollment, BR023 had metastases in multiple organs, including the brain, liver, and bone. After multiple cycles of dual HER2-targeted therapy and chemotherapy, ^99m^Tc-NM-02 SPECT/CT showed low uptake in the metastases, except for those in the brain, implying ineffective treatment for these brain lesions. Despite ^18^F-FDG uptake being at the background level in the metastases during the subsequent HER2-targeted therapy, brain metastases had grown in number and expressed higher ^18^F-FDG uptake.

**Fig. 5:**
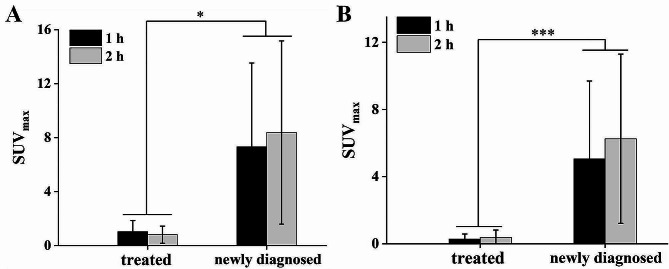
Differences in ^99m^Tc-NM-02 uptake in primary lesions (**A**) and metastases (**B**) among three newly diagnosed patients with HER2-positive breast cancer and four previously treated patients with HER2-positive breast cancer after multiple cycles of HER2-targeted therapy

Notably, among the seven patients whose HER2-targeted therapy was effective, three were newly diagnosed, and four had received multiple cycles of HER2-targeted therapy before enrollment. Comparing the SUV_max_ of the lesions in these patients, the ^99m^Tc-NM-02 uptake in the lesions of the four previously-treated patients was lower than that in the three newly diagnosed patients (Fig. 5). Moreover, the contemporaneous ^18^F-FDG PET/CT showed low uptake in the lesions of four treated patients, and similar results were also found in the follow-up ^18^F-FDG PET/CT imaging of the three newly diagnosed patients after multiple cycles of HER2-targeted therapy.

## Discussion

The importance of the accurate detection of HER2 expression is widely recognized in clinical practice. With a deeper understanding of the HER2 protein, the definition and interpretation of HER2 positivity have been revised several times to facilitate the establishment of standardized tests which produce accurate results [25]. However, the high mortality rate of breast cancer has prompted the development of novel molecular imaging techniques for accurate HER2 detection [11]. Although radiolabeled anti-HER2 antibodies, such as ^89^Zr-trastuzumab or ^111^In-pertuzumab, show high potential for HER2 molecular imaging, they may not be the best for clinical applications because of their poor pharmacokinetic characteristics and slow tumor uptake [12]. In contrast to large intact antibodies, other candidates with small size, including antibody fragments, affibodies, and peptides, have shorter circulation times, deeper tumor penetration, and are suitable for radiolabeling with short half-life radionuclides, making them superior to conventional antibodies in the establishment of HER2-targeted probes for molecular imaging. Many successful preclinical and clinical studies of these radiolabeled candidates have demonstrated the feasibility of targeted detection of tumor HER2 expression [12–14, 26–31]. Meanwhile, several clinical trials are currently underway using HER2-targeted molecular probes to noninvasively assess HER2 status or screen patients who can benefit from HER2 target treatment (including but not limited to NCT05535621, NCT04547309 and NCT03655353). In our previous study, we developed a ^99m^Tc-labeled single-domain antibody NM-02 as a SPECT probe for non-invasive assessment of HER2 expression. We demonstrated its good safety, acceptable radiation dosimetry, and favorable biodistribution in 10 patients with breast cancer [23]. Based on those data, 30 patients with breast cancer were recruited in this study to further evaluate the clinical value of ^99m^Tc-NM-02 in visualizing whole-body HER2 expression and heterogeneity.

Considering the unevenness of ^99m^Tc-NM-02 uptake in lesions, for quantitative analysis in this study, we used both SUV_max_ and SUV_mean_, which show the maximum and overall uptake of ^99m^Tc-NM-02, respectively [20]. Moreover, depending on whether they had received antitumor treatment before enrollment, 30 patients were divided into the newly diagnosed and treated groups. This grouping method permitted analyzing the correlation between ^99m^Tc-NM-02 uptake and HER2 expression and evaluating the changes in tumor expression of HER2 after treatment [32, 33]. In the newly diagnosed group, both the SUV_max_ and SUV_mean_ in the primary and metastatic lesions were well correlated with HER2 status, and the SUV_max_ and SUV_mean_ in the HER2-positive group were significantly higher than those in the HER2-negative group. Notably, ^99m^Tc-NM-02 uptake by lesions in the newly diagnosed group increased as HER2 IHC results progressed. However, the deviation of SUV_max_ and SUV_mean_ seemed large, which may be related to the failure to capture the region with a high uptake of ^99m^Tc-NM-02 through puncturing, resulting in a mismatch between HER2 IHC and ^99m^Tc-NM-02 uptake. Meanwhile, partial volume correction was not performed in this study, which might also be a factor for the large deviation of SUVs. Therefore, further studies of ^99m^Tc-NM-02 SPECT/CT imaging-guided biopsy for non-invasive quantification of HER2 expression are required to determine the relationship between HER2 expression measured by partial volume corrected ^99m^Tc-NM-02 SPECT/CT and pathological results from tumor biopsies.

In the treated group, ^99m^Tc-NM-02 uptake in the primary and metastatic lesions was inconspicuous, with no significant difference between HER2-positive and HER2-negative patients, and the SUV_max_ and SUV_mean_ were significantly lower than those in the newly diagnosed HER2-positive patients. Similarly, this tendency was also observed in seven HER2-positive patients who benefited from follow-up HER2-targeted therapy. ^99m^Tc-NM-02 uptake in the lesions of the four patients who had previously received drug treatment was significantly lower than that of the three newly diagnosed patients, indicating that HER2-targeted therapy reduces the expression of HER2 or the binding activity of ^99m^Tc-NM-02 against HER2 protein [34]. ^99m^Tc-NM-02 SPECT/CT can potentially be used to monitor and evaluate the efficacy of HER2-targeted therapy. When trastuzumab binds to the epitope of the HER2 receptor, the antibody receptor complex is internalized into tumor cells by endocytosis, blocking HER2 protein from reaching the cell surface and accelerating its degradation. HER2 expression decreases with the low uptake of ^99m^Tc-NM-02 when trastuzumab treatment is effective in HER2-positive lesions. In contrast, the lack of a significant decrease in ^99m^Tc-NM-02 uptake in the lesion during the therapy process may suggest poor efficacy of HER2-targeted therapy. This view was supported by the high uptake of ^99m^Tc-NM-02 in the brain and multiple bone metastases after several cycles of HER2-targeted therapy, consistent with subsequent disease progression. Nevertheless, a longitudinal comparison between pre- and post-treatment ^99m^Tc-NM-02 SPECT/CT imaging should be performed to further test this hypothesis. Bedsides, the relatively small sample size in the treated group (*n* = 6) may be a limitation, and larger cohorts have been planned in future longitudinal studies.

Non-specific uptake in inflammatory tissues has always been a shortcoming of ^18^F-FDG PET/CT in clinical applications. Eliminating the interference of inflammatory tissues is conducive to more accurate imaging results and strongly supports the specificity of ^99m^Tc-NM-02. As expected, the uptake of ^18^F-FDG at the inflammation and tumor sites was significantly high in the three patients diagnosed with chest inflammation, while ^99m^Tc-NM-02 only accumulated in the tumors. The effectiveness of subsequent anti-inflammatory therapy for these patients displayed high consistency with ^18^F-FDG and ^99m^Tc-NM-02 diagnoses, further confirming that ^99m^Tc-NM-02 SPECT/CT results could not be disturbed by inflammatory tissues. However, drawing an immediate conclusion from the data of three patients is challenging, and more evidence from a larger sample size is needed. Because of the diversity of inflammation, only chest inflammation was evaluated in this study, and the void uptake of ^99m^Tc-NM-02 in other types of inflammatory tissues also remains to be verified.

Heterogeneous HER2 expression in breast cancer has frequently been described, and radionuclide molecular imaging of HER2 holds great potential to avoid biopsy with bias from tumor heterogeneity [11]. Therefore, visualizing the heterogeneity of HER2 expression in 30 patients with breast cancer was the primary purpose of this study. As expected, ^99m^Tc-NM-02 SPECT/CT revealed uneven uptake in nine patients, including heterogeneous intratumoral uptake and inconsistent uptake in multiple unilateral lesions, bilateral primary lesions, or between primary and metastatic lesions, suggesting intra- and intertumoral heterogeneity of HER2 expression. Notably, this tendency was observed in patients with multiple or large lesions, generally unsuitable for surgical treatment or neoadjuvant chemotherapy followed by surgery. Comprehensive HER2 detection by ^99m^Tc-NM-02 SPECT/CT may help screen out HER2-positive lesions in these patients and potentially change their therapeutic strategies to benefit from HER2-targeted drugs.

Other research groups have explored PET/CT imaging strategies using ^68^Ga-labeled nanobodies to assess HER2 expression [20]. The phase I study data showed good safety, favorable biodistribution, reasonable dosimetry, and satisfactory tumor-targeting potential, similar to our first-in-human study of ^99m^Tc-NM-02 [23]. In addition, a phase II trial of the ^68^Ga-labeled nanobody to assess the relationship between PET/CT and IHC using a semi-quantitative scale is ongoing [22]. In this study, a good relationship between ^99m^Tc-NM-02 SPECT/CT and pathological results was found in newly diagnosed patients with breast cancer. These findings may be a catalyst for other novel HER2-targeted probes. In combination with the rapid development of radionuclide therapy, the uptake of ^99m^Tc-NM-02 in HER2-positive lesions also facilitates the targeted therapeutic potentials of NM-02. This nanobody after radiolabeling with therapeutic radionuclides, such as ^177^Lu, ^188^Re, or ^131^I, may provide new therapeutic options for HER2-positive patients. Based on the results of this study, we will continue clinical investigations by using other radionuclide-labeled NM-02 for potential treatments in patients with HER2-positive breast cancer.

## Conclusions

The uptake of ^99m^Tc-NM-02 correlates well with HER2 expression in newly diagnosed patients with breast cancer. The low ^99m^Tc-NM-02 accumulation in primary lesions and metastases in the treated group is probably related to the influence of treatment. ^99m^Tc-NM-02 shows no non-specific uptake in inflammatory tissues and good performance for detecting brain and bone metastases from HER2-positive breast cancer. Heterogeneity of HER2 expression in breast cancer can be visualized and revealed by ^99m^Tc-NM-02 SPECT/CT. ^99m^Tc-NM-02 SPECT/CT has high potential to be a comprehensive and promising method for evaluating HER2 expression.

### Electronic supplementary material

Below is the link to the electronic supplementary material.

Supplementary Material 1

## Data Availability

The datasets used and analyzed during the current study are available from the corresponding author on reasonable request.

## Abbreviations

^18^F-FDG: ^18^F-fluorodeoxyglucose
^99m^Tc-NM-02: ^99m^Tc radiolabeled nanobody NM-02
CT: computed tomography
FISH: fluorescence in situ hybridization
HER2: human epidermal growth factor receptor 2
IHC: immunohistochemistry
PET: positron emission tomography
SPECT: single photon emission computed tomography
SUV_max_: maximal standard uptake value
SUV_mean_: mean standard uptake value
