# Supplementary material for: Detection of HER2 expression using 99mTc-NM-02 nanobody in patients with breast cancer: a non-randomized, non-blinded clinical trial
Source: Breast Cancer Res. 2024 Mar 8;26:40. doi: 10.1186/s13058-024-01803-y (PMC10924314; doi:10.1186/s13058-024-01803-y)
Supplement: Supplementary file 1 — Supplementary Material 1 [file 13058_2024_1803_MOESM1_ESM.docx]

**Supplementary Material**

**Supplementary Table S1****. Patient characteristics**

| Patient number | Age (years) | Weight (Kg) | ^99m^Tc-NM-02 dose (MBq) | ^18^F-FDG dose (MBq) | Tumor  type | ER/PR | IHC | FISH | Primary tumor position | Metastatic lesion | Clinical staging |
| --- | --- | --- | --- | --- | --- | --- | --- | --- | --- | --- | --- |
| BR011 | 32 | 65 | 535.02 | 286.01 | IPC | +/+ | 2+ | - | right | LNM | T2N1M0 |
| BR012 | 53 | 57.5 | 498.02 | 222.0 | IDC | -/- | 3+ | + | right | LNM, LM, BM | T2N2M1 |
| BR013 | 48 | 79 | 572.02 | 262.7 | IPC | -/- | 3+ | + | right | LNM, LM, BM | T2N3M1 |
| BR014 | 29 | 56 | 381.1 | 218.3 | left IPC  right IDC | left +/+  right +/+ | left 0  right 2+ | left -  right - | bilateral | / | left T2N0M0  right T2N0M0 |
| BR015 | 72 | 61 | 500.24 | 229.4 | ILC | -/- | 0 | - | left | LNM, BM | T2N3M1 |
| BR016 | 46 | 58.5 | 467.051 | 263.07 | IDC | -/- | 3+ | + | left | LNM | T2N1M0 |
| BR017 | 64 | 45 | 459.54 | 243.46 | IDC | +/- | 3+ | + | left | LNM | T2N1M0 |
| BR018 | 41 | 68 | 437.71 | 277.5 | up IDC  down IDC | up +/+  down +/+ | up 1+  down 2+ | up -  down - | left | / | T2N0M0 |
| BR019 | 64 | 60 | 435.49 | 244.94 | IDC | +/+ | 2+ | + | left | LNM | T1N3M0 |
| BR020 | 67 | 60.5 | 446.96 | 288.6 | IPC | +/+ | 1+ | - | left | / | T2N0M0 |
| BR021 | 50 | 88 | 578.31 | 362.6 | IPC | +/+ | 2+ | - | right | LNM | T2N1M0 |
| BR022 | 34 | 53 | 529.1 | 215.71 | IPC | -/- | 1+ | - | right | / | T2N0M0 |
| BR023 | 49 | 48 | 619.38 | 196.47 | IDC | -/- | 3+ | + | right | LNM, LM, BM, CM | T3N3M1 |
| BR024 | 55 | 65 | 576.46 | 265.29 | IPC | -/- | 3+ | + | right | / | T2N0M0 |
| BR025 | 27 | 47.5 | 442.15 | 191.66 | IPC | -/- | 1+ | - | right | / | T1N0M0 |
| BR026 | 76 | 63 | 559.44 | 253.08 | IPC | +/+ | 1+ | - | right | / | T1N0M0 |
| BR027 | 64 | 76 | 461.39 | 281.2 | IPC | +/+ | 3+ | + | right | / | T2N0M0 |
| BR028 | 68 | 45.1 | 559.44 | 187.96 | IPC | -/- | 1+ | - | right | LNM | T1N2M0 |
| BR029 | 53 | 65 | 370.74 | 262.7 | MBC | +/+ | 0 | - | left | / | T2N0M0 |
| BR030 | 46 | 67 | 574.98 | 281.2 | IPC | -/- | 0 | - | left | LNM | T2N2M0 |
| BR031 | 55 | 52.5 | 526.51 | 203.5 | IDC | +/- | 2+ | + | left | / | T2N0M0 |
| BR032 | 39 | 73.5 | 536.87 | 303.4 | IPC | -/- | 0 | - | right | / | T1N0M0 |
| BR033 | 43 | 72 | 512.08 | 268.99 | IPC | +/- | 2+ | - | right | LNM | T1N2M0 |
| BR034 | 43 | 66 | 427.35 | 247.9 | IPC | +/+ | 1+ | - | left | / | T1N0M0 |
| BR035 | 57 | 55 | 537.61 | 203.13 | IPC | +/- | 1+ | - | left | LNM | T1N2M0 |
| BR036 | 57 | 65 | 479.15 | 238.28 | IPC | +/+ | 2+ | - | left | LNM | T2N3M0 |
| BR037 | 59 | 70 | 437.34 | 260.11 | IPC | -/- | 1+ | - | right | / | T2N0M0 |
| BR038 | 50 | 67.2 | 360.75 | 270.1 | IPC | -/- | 0 | - | right | LNM | T2N1M0 |
| BR039 | 48 | 48.4 | 411.44 | 192.4 | IPC | +/+ | 1+ | - | right | LNM | T2N1M0 |
| BR040 | 66 | 62 | 508.75 | 307.1 | IPC | -/- | 3+ | + | right | LNM | T1N3M0 |

ER = estrogen receptor; PR = progesterone receptor; IPC = invasive papillary carcinoma; IDC = invasive ductal carcinoma; ILC = invasive lobular carcinoma; MBC = mucinous breast carcinoma; LNM = lymph node metastasis; BM = bone metastasis; LM = liver metastasis; CM = cerebral metastasis; Clinical staging was determined using the eighth edition of the American Joint Committee on Cancer staging for breast cancer. Metastases were confirmed after a thorough review of imaging and pathological findings. There were 32 primary tumors in 30 patients owing to the double primary lesions with different HER2 expression levels in BR014 and in BR018. LNM, BM, CM and LM were respectively found in 17, three, one, and two patients with breast cancer. Twenty-four breast cancer patients were newly diagnosed, and the remaining six patients (BR012, BR013, BR021, BR023, BR033 and BR038) had received multiple cycles of targeted therapies or chemotherapy before enrollment.

**Supplementary Table S2. Analyses of ^18^F-FDG PET/CT and ^99m^Tc-NM-02 SPECT/CT imaging findings**

| Thirty patients with pathologically confirmed breast cancer | | | | Primary lesion | LNM | BM | LM | CM |
| --- | --- | --- | --- | --- | --- | --- | --- | --- |
|  |  |  |  | 32 | 17 | 3 | 2 | 1 |
| Positive imaging by visual interpretation / number of patients | ^18^F-FDG | | | 32/32 | 25/17 | 3/3 | 2/2 | 1/1 |
|  | ^99m^Tc-NM-02 | Newly diagnosed group | IHC 0 | 1/5 | 2/2 | 1/1 |  |  |
|  |  |  | IHC 1 | 8/10 | 3/3 |  |  |  |
|  |  |  | IHC 2 | 4/4 | 2/2 |  |  |  |
|  |  |  | IHC 3 | 7/7 | 3/4 |  |  |  |
|  |  | Treated group | | 2/6 | 0/6 | 0/2 | 0/2 | 1/1 |
| SUV_max_ (1 h) | ^18^F-FDG | | | 10.09 ± 9.02 | 5.54 ± 4.60 | 8.50 ± 1.11 | 14.01 ± 7.78 | 14.85 ± 2.49 |
|  | ^99m^Tc-NM-02 | Newly diagnosed group | IHC 0 | 1.16 ± 1.13 | 3.40 ± 1.56 | 8.21 ± 1.06 |  |  |
|  |  |  | IHC 1 | 3.56 ± 2.44 | 4.25 ± 2.64 |  |  |  |
|  |  |  | IHC 2 | 5.01 ± 2.26 | 2.61 ± 0.70 |  |  |  |
|  |  |  | IHC 3 | 6.52 ± 5.12 | 6.01 ± 4.81 |  |  |  |
|  |  | Treated group | | 1.78 ± 1.98 | 0.72 ± 0.55 | 0.65 ± 0.52 | 0.69 ± 0.53 | 0.62 ± 0.52 |
| SUV_max_ (2 h) | ^99m^Tc-NM-02 | Newly diagnosed group | IHC 0 | 1.10 ± 1.02 | 4.40 ± 3.03 | 7.81 ± 0.94 |  |  |
|  |  |  | IHC 1 | 3.50 ± 2.63 | 4.22 ± 3.08 |  |  |  |
|  |  |  | IHC 2 | 5.25 ± 1.82 | 3.54 ± 2.46 |  |  |  |
|  |  |  | IHC 3 | 7.46 ± 5.65 | 7.55 ± 4.84 |  |  |  |
|  |  | Treated group | | 1.88 ± 2.45 | 0.50 ± 0.45 | 0.50 ± 0.49 | 0.48 ± 0.52 | 0.38 ± 0.43 |

**Supplementary Table S3.** Changes of HER2 expression before and after treatment

| Patient number | Percutaneous biopsy | HER2-targeted therapy | Postoperative pathology |
| --- | --- | --- | --- |
| BR017 | 3+ | + | 2+ |
| BR019 | 2+ | - | 1+ |
| BR020 | 1+ | - | 2+ |
| BR022 | 1+ | - | 0 |
| BR037 | 1+ | - | 0 |
| BR038 | 0 | - | 1+ |

**
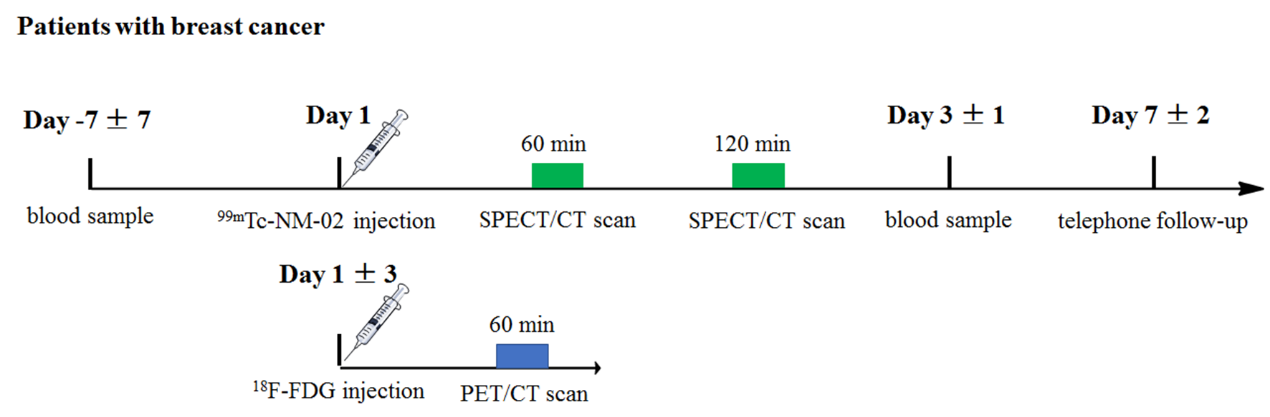
**

**Supplementary Figure S1.** Timelines for the ^99m^Tc-NM-02 SPECT/CT and ^18^F-FDG PET/CT imaging study in 30 patients with breast cancer. Blood samples were collected from patients within 14 days before enrollment and after 3 days of ^99m^Tc-NM-02 injection. Telephone follow-up was carried out after 7 days of ^99m^Tc-NM-02 injection. SPECT/CT imaging was performed at 1 and 2 h after ^99m^Tc-NM-02 injection. PET/CT imaging was performed at 1 h after ^18^F-FDG injection.


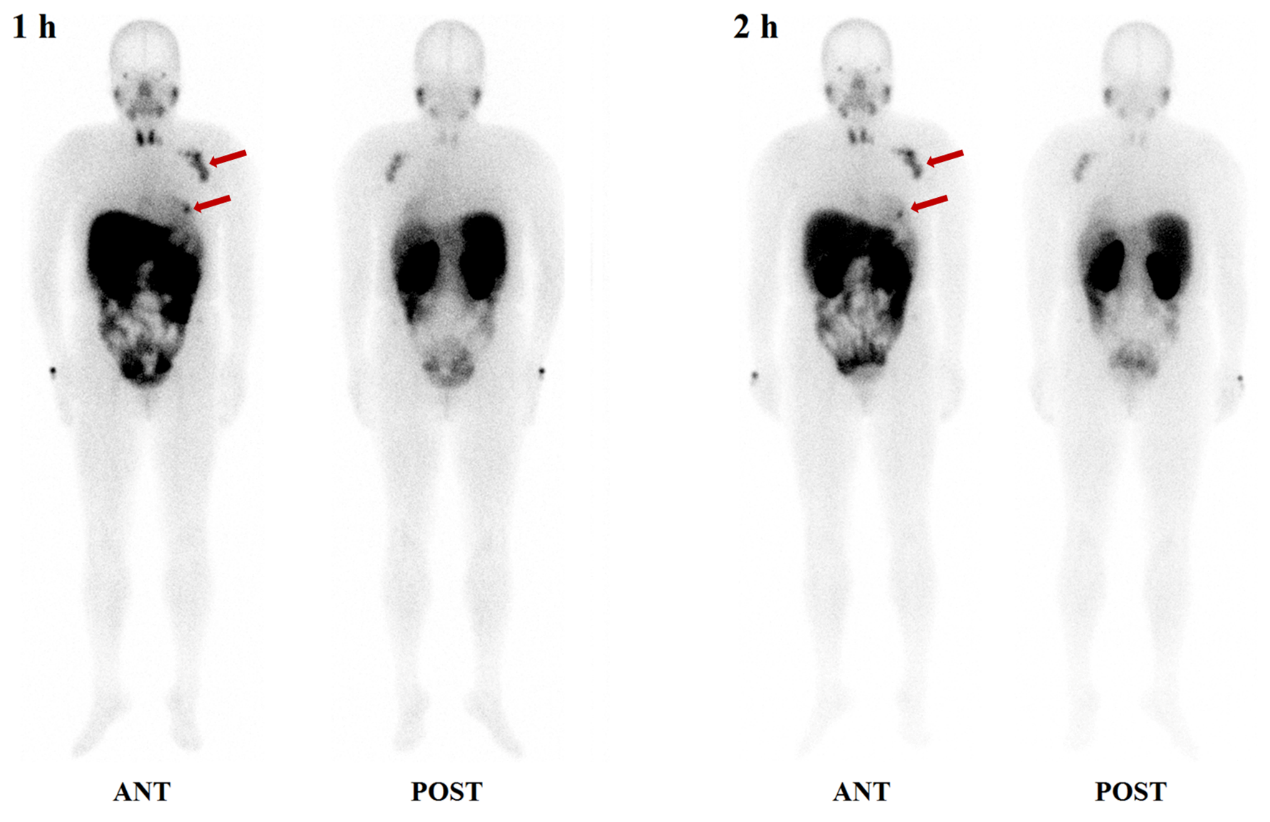


**Supplementary Figure S2.** Anterior (ANT) and posterior (POST) whole-body ^99m^Tc-NM-02 SPECT images of BR019 (HER2 IHC 2+, FISH +) at 1 and 2 h post-injection. The red arrows represent the primary lesion in the right breast and lymph node metastases in the right axilla.


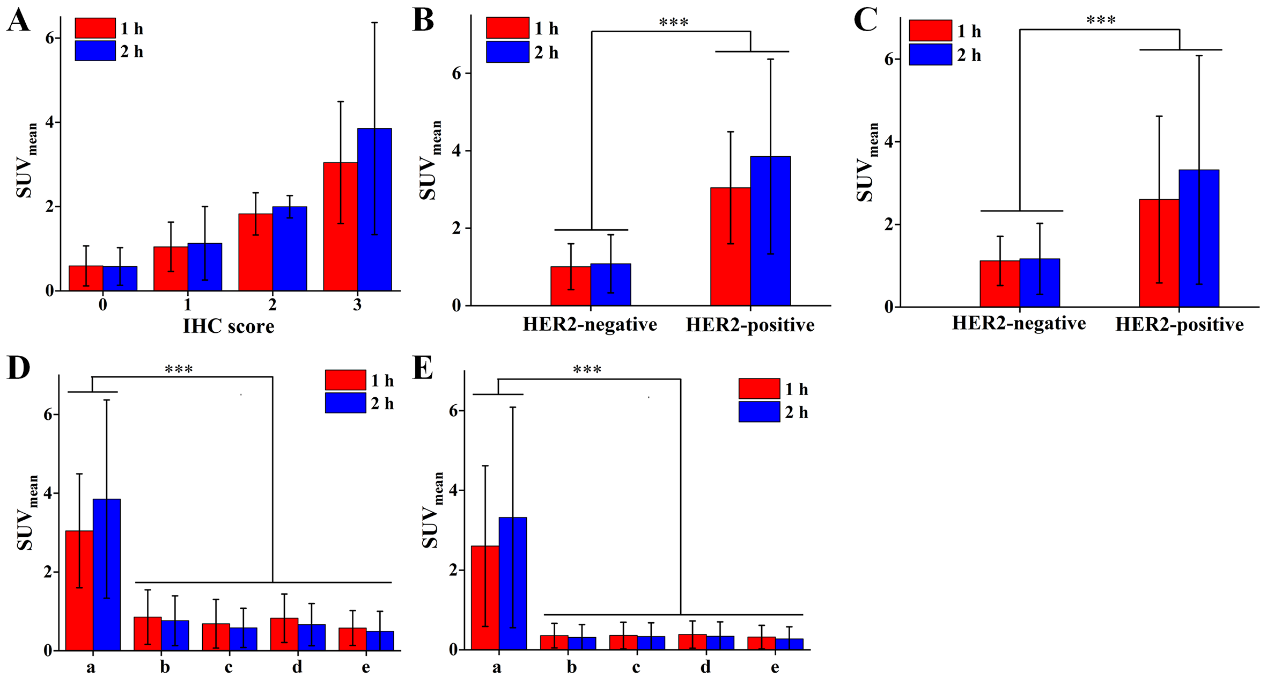


**Supplementary Figure S3.** Relationship between ^99m^Tc-NM-02 SUV_mean_ and HER2 IHC scores in the primary lesions of the newly diagnosed group (A). Correlations were found at 1 h (r^2^ = 0.956, P = 0.021) and 2 h post-injection (r^2^ = 0.925, P = 0.037). Relationship between ^99m^Tc-NM-02 SUV_mean_ and HER2 status in the primary lesions (B) and metastases (C) of the newly diagnosed group. Relationship between ^99m^Tc-NM-02 SUV_mean_ and HER2 status in the primary lesions (D) and metastases (E) of the treated group; a: seven HER2-positive patients in the newly diagnosed group, b: six patients in treated group, c: five patients with high HER2 expression (HER2 2+ and 3+) in treated group, d: four HER2-positive patients in treated group, e: three HER2-positive patients with effective HER2-targeted therapy in treated group.


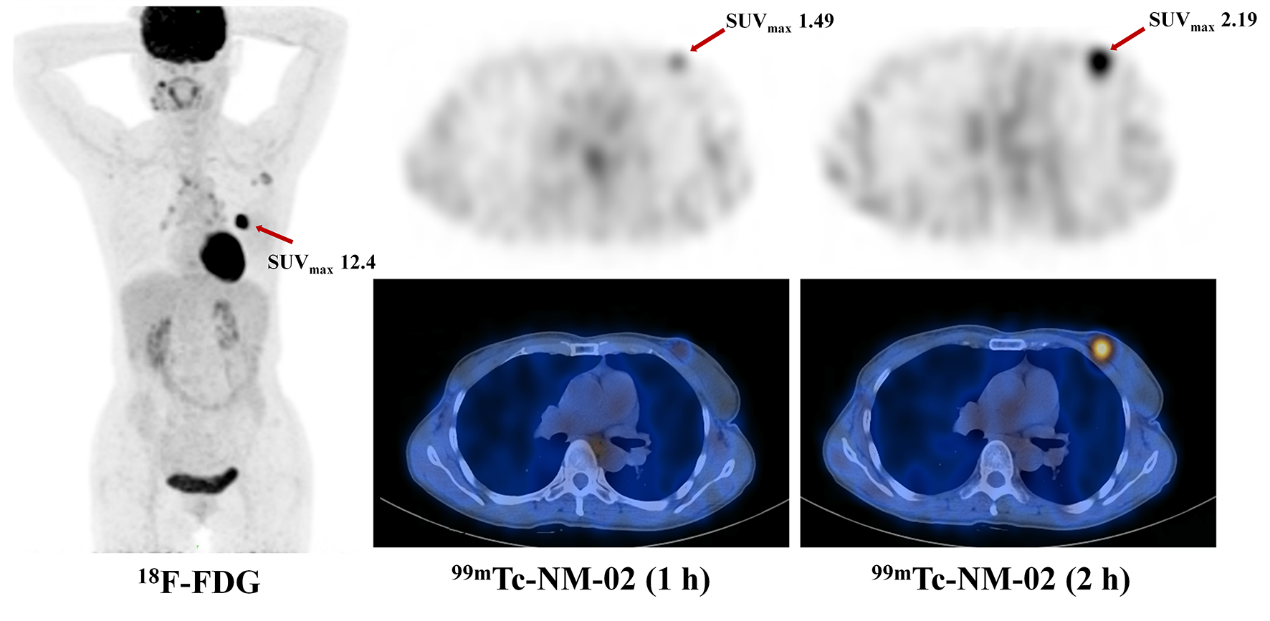


**Supplementary Figure S4.** ^18^F-FDG PET/CT and ^99m^Tc-NM-02 SPECT/CT of BR017 (HER2 3+). The visual interpretation of BR017 (HER2 3+) was negative at 1 h post-injection, but positive at 2 h, which was consistent with the corresponding SUV_max_ (1.49 vs 2.19).

**
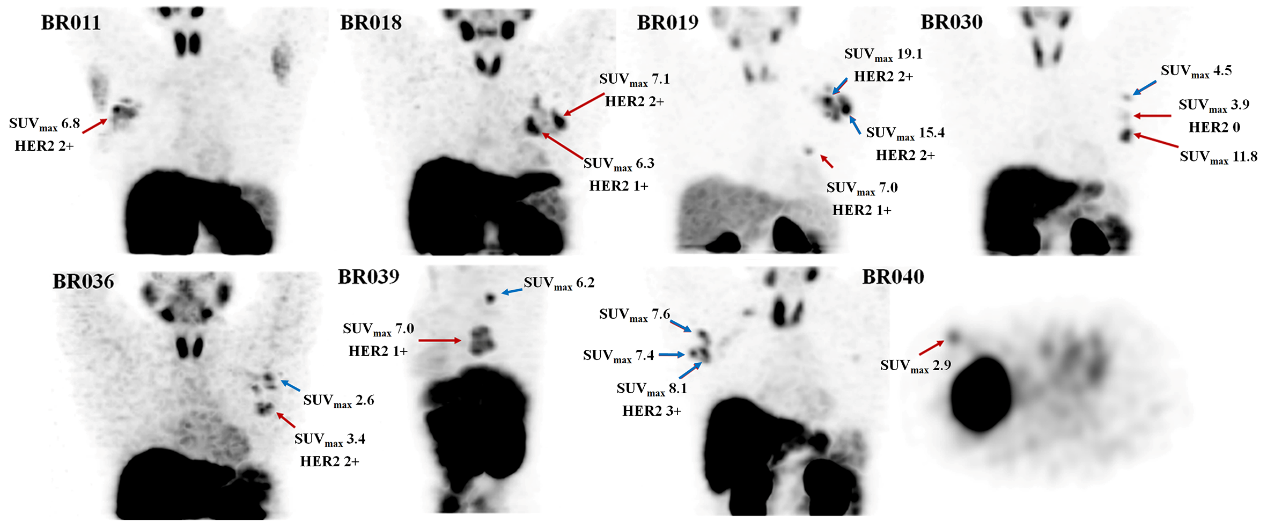
**

**Supplementary Figure S5.** Heterogeneity of HER2 expression by ^99m^Tc-NM-02 SPECT/CT imaging. BR011, BR036 and BR039 had uneven uptake of ^99m^Tc-NM-02 within the primary lesions. BR018 and BR030 showed inconsistent uptake of ^99m^Tc-NM-02 in multiple unilateral lesions. BR019 and BR040 displayed heterogeneous uptake of ^99m^Tc-NM-02 between primary and metastatic lesions. Red and blue arrows respectively represent the primary lesions and metastases.


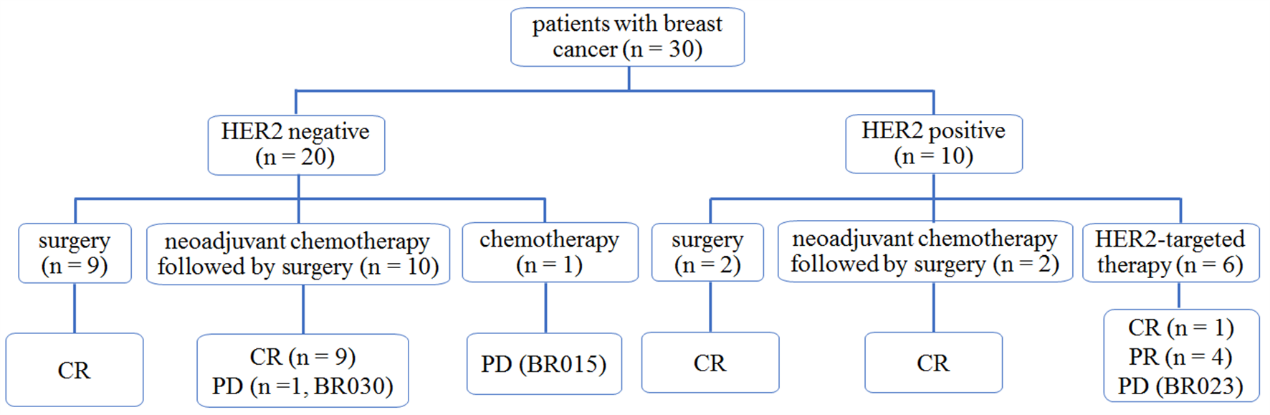


**Supplementary Figure S6.** Clinical outcomes of 30 patients with breast cancer after treatment. Eighteen (18/20) HER2-negative patients were complete response (CR) and two patients (BR015 and BR030) had progressive disease (PD). Five (5/10) HER2-positive patients were CR, four patients were partial response (PR) and one patient (BR023) had PD.

**
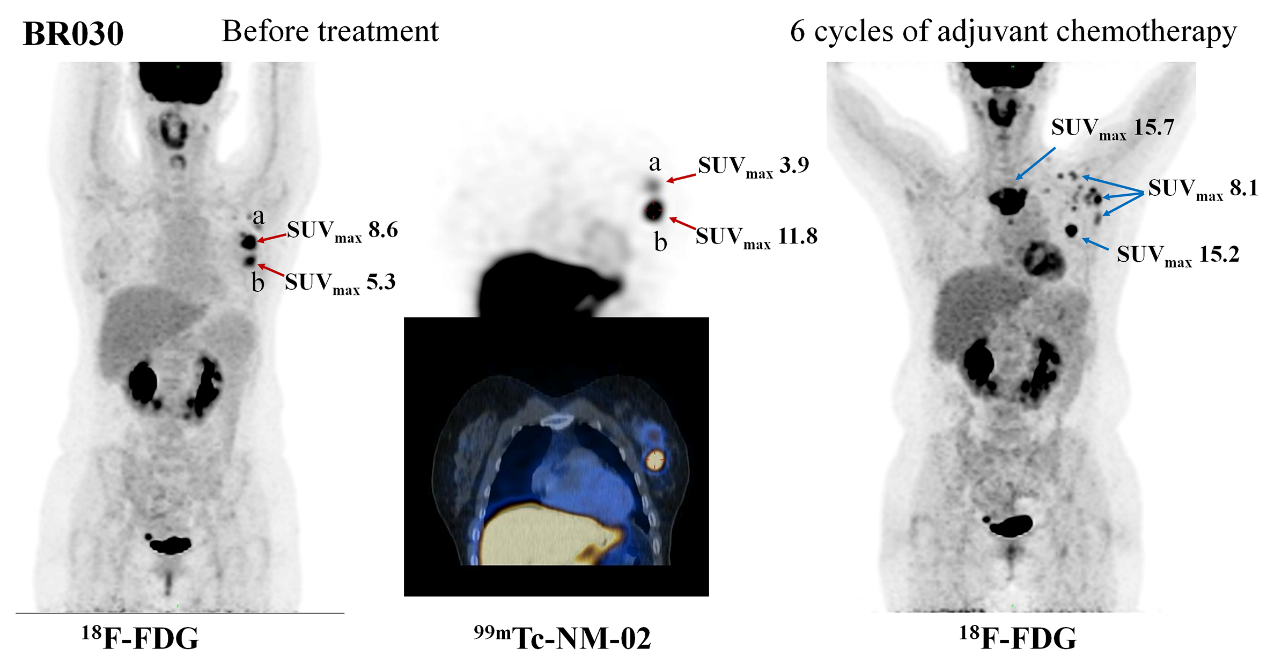
**

**Supplementary Figure S7.** ^18^F-FDG PET/CT and ^99m^Tc-NM-02 SPECT/CT of BR030 (HER2 IHC 0) at 1 h post-injection. BR030 had multiple lesions in the left breast, and only the larger lesion with high ^18^F-FDG but low ^99m^Tc-NM-02 uptake (lesion a) was punctured to determine the HER2 status, while the ^99m^Tc-NM-02 uptake in the smaller lesion (lesion b) was high, suggesting potential HER2-positive lesions. After 6 cycles of postoperative adjuvant chemotherapy (pirarubicin–cyclophosphamide), ^18^F-FDG PET/CT suggested disease progression with lymph node and bone metastases.

**
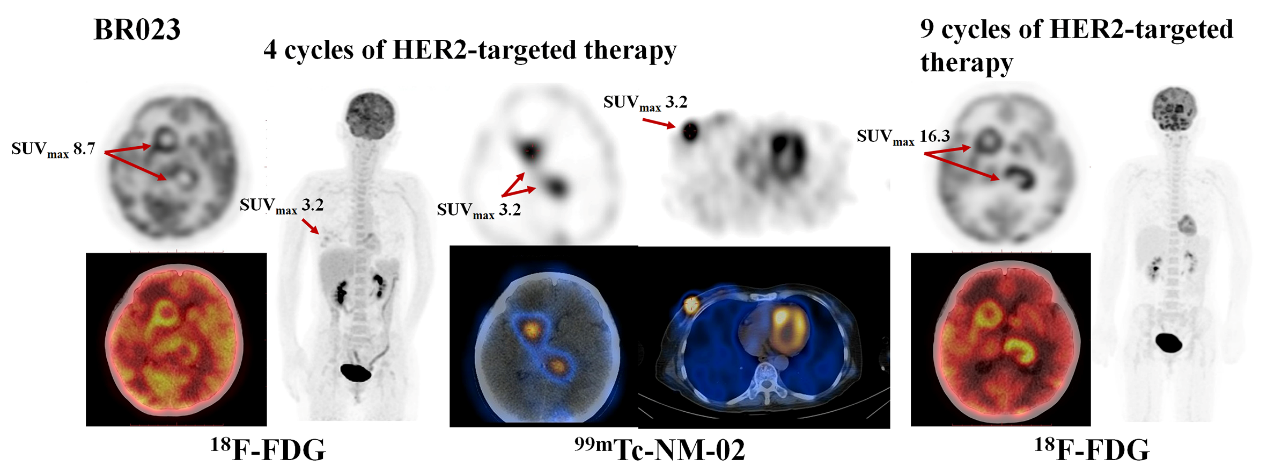
**

**Supplementary Figure S8.** ^18^F-FDG PET/CT and ^99m^Tc-NM-02 SPECT/CT of BR023 at 1 h post-injection. BR023 had metastases in multiple organs including brain, liver and bone before enrollment. After 4 cycles of trastuzumab–pertuzumab–docetaxel, ^99m^Tc-NM-02 SPECT/CT showed mild accumulation in the primary lesion and low uptake in the metastases except the brain metastases, implying ineffective treatment for these brain lesions. Despite the ^18^F-FDG uptake was background level in the primary lesions and metastases after 9 cycles of HER2-targeted therapy, brain metastases had grown with more lesions and higher ^18^F-FDG uptake.
